# Quack Medicines

**Published:** 1910-04-16

**Authors:** Eva Richmond

**Affiliations:** Rockhampton, Falfield, Glos.


*To the Editor of* The Hospital.

### QUACK MEDICINES.

*To the Editor of* The Hospital.

Sir,—In your issue of February 12 you have a note on the subject of *Truth's* Caution List. May I be allowed to say a word or two on the matter?

I have no doubt the Caution List is very useful, but it seems to me that it does not quite meet the need, and this by reason of its price. The people most likely to be led away by the lying—and sometimes almost indecent—"puffs" of patent medicines are the poor, who certainly cannot afford to pay 1s. for the Caution List. Besides, the ignorant reader of the halfpenny daily, who wastes his money, time, and health on the quack remedies advertised therein, has probably never heard of *Truth's* exposure of these remedies. Further, that the doctor possesses a copy of the Caution List does not help the poor victim, as he buys and swallows the rubbish in order to avoid having to send for a medical man, thinking it cheaper to go on spending small sums in the purchase of boxes of pills than to get good advice, though the latter is, of course, much cheaper in the end.

May I suggest that what is really needed is cheap leaflets which should expose these lying quacks—leaflets which one could buy at 50 for 1s., or something like that, for distribution. Personally I would gladly get and distribute leaflets exposing these quacks—it angers me to think of all the harm they do, and all the suffering they cause to the poor people they take in—but I cannot afford to do this with a publication of which each copy costs 1s. Could not some medical paper—your own, for instance—or society be enterprising enough to do this? I did suggest it to the Editor of *Truth*, but he did not see it; but could not some medical paper rise to the occasion?

Yours faithfully,

(Miss) Eva Richmond.

Rockhampton, Falfield, Glos.

